# '*In Every River, in Every Lake*': Impacts of Beaver Population Change on Indigenous Livelihoods and Wellbeing in the Inuvialuit Settlement Region, Western Canadian Arctic

**DOI:** 10.1007/s10745-026-00758-2

**Published:** 2026-09-03

**Authors:** Callum Pearce, Melanie Rohse, Nicola Walshe, Helen C. Wheeler

**Affiliations:** 1 Anglia Ruskin University, Cambridge, UK; 2 University College London, London, UK

**Keywords:** Inuvialuit, Canadian Arctic, Beaver, *Castor canadensis*, Indigenous peoples, Wellbeing

## Abstract

Ecosystem transformation driven by climatic, environmental and societal factors poses interlinked ecological and social challenges for many Indigenous communities. For Inuvialuit communities in and around the lower Mackenzie Delta, in the western Canadian Arctic, recent changes in the population of beavers (*Castor canadensis*) have disrupted subsistence harvesting activities and travelling routes, exacerbating stresses linked to the cost of living and increasing the exposure of active harvesters to risk. Drawing on interviews with 25 Inuvialuit knowledge holders from the three communities of Inuvik, Tuktoyaktuk, and Aklavik, we investigated concerns around beaver population change in the western Arctic and the impact of this change on Inuvialuit livelihoods and wellbeing. Beyond the direct impact of beavers, the issues associated with ecosystem transformation pose challenges for Inuvialuit collective identity, the continuity of knowledge and connections to the land. While beaver population change was widely attributed to the local effects of a warming climate, participants also pointed to the effects of social transformations over recent decades. Ecological changes interact with social factors to produce complex and wide-ranging consequences for Indigenous communities.

## Introduction

The Arctic is transforming as the climate warms. Across Arctic Alaska and Inuit Nunangat (the land, water, and ice that make up the Inuit homeland in Canada), higher temperatures have driven permafrost thaw (Romanovsky et al., 2010; Liljedahl et al., 2016), led to longer summers with earlier ice break-up and shorter periods of snow cover (Smejkalová et al., 2016; St. Jacques & Sauchyn, 2009; Surdu et al., 2014), and contributed in some regions to the shrubification of tundra through an increase in the growth and abundance of deciduous shrub vegetation (Sturm et al., 2001; Lantz et al., 2009; Myers-Smith et al., 2011; Xu et al., 2013).

These biophysical processes may be driving ecosystem regime shifts marked by species distribution change and further accompanying biophysical transformations. The most extreme forms of ecosystem change are regime shifts, where ecosystems shift to new states governed by new sets of feedbacks and processes (Andersen et al., 2009). Under climate change, the frequency of regime shifts is increasing (Folke, 2006; Rocha et al., 2015; Scheffer et al., 2015). Understanding how they progress and what determines transitions to alternative stable states is key to making informed stewardship decisions for building resilience to the impacts of rapidly changing environments. More recent thinking concerning regime shifts has expanded beyond traditional systems thinking to incorporate empirical realisations of change and the social processes that produce our understandings of those realities (Kull et al., 2018).

The impacts of these biophysical and environmental shifts exemplify the interlinkages between rapid climate change, ecosystem change, and social processes, helping us better understand the risks associated with rapid change. With warming in the Arctic occurring at nearly four times the global average since 1979 (Rantanen et al., 2022), this region may provide early indications of the nature of societal impacts likely to emerge globally as a result of climate change. One potential route to regime shifts in the Arctic is borealisation: the northward expansion of species of boreal forest plants and wildlife (Tape et al., 2016). Borealisation has been observed in both terrestrial and marine environments (Fossheim et al., 2015; Verdonen et al., 2026), and has the potential to create a suite of societal impacts (Baker et al., 2025). Notable features of observed changes have included impacts on transportation, infrastructure, and Indigenous food security and sovereignty, with major implications for Indigenous livelihoods and cultural activities (Ford et al., 2021), as well as impacts on sense of place, physical and emotional health, and wellbeing (Cunsolo Willox et al., 2012). This has driven a focus on adaptive strategies and socio-ecological resilience (Huntington et al., 2018).

Species involved in borealisation include the North American beaver (*Castor canadensis*). Often referred to as ecosystem engineers, beavers physically engineer changes in their environments and may amplify the societal impacts of warming. Beavers appear to be increasing in population density and expanding their distribution in tundra regions north of the treeline across the western North American Arctic (Hole et al., 2026). This distribution change may be a response to increased shrub growth and longer periods of unfrozen water, driven by warming (Tape et al., 2018). This region has also seen major changes in the intensity of hunting and trapping activity over the past 70 years, however, which may be another driver of beaver population growth (Krause, 2023). Beavers have the potential to affect freshwater and riparian ecosystems: beaver dams create new ponds and channels, changing the water level and disrupting the flow of existing streams, which may in turn disrupt the movement of fish. These activities may exacerbate the local effects of warming in the Arctic, accelerating permafrost thaw (Tape et al., 2022; Glass et al., 2025). As ecosystem regime shifts have the potential to impact human livelihoods and wellbeing (Shackleton et al., 2018), beaver population change may also impact Indigenous communities engaged in subsistence harvesting (hunting, trapping, fishing, and berry-picking).

**Fig. 1:**
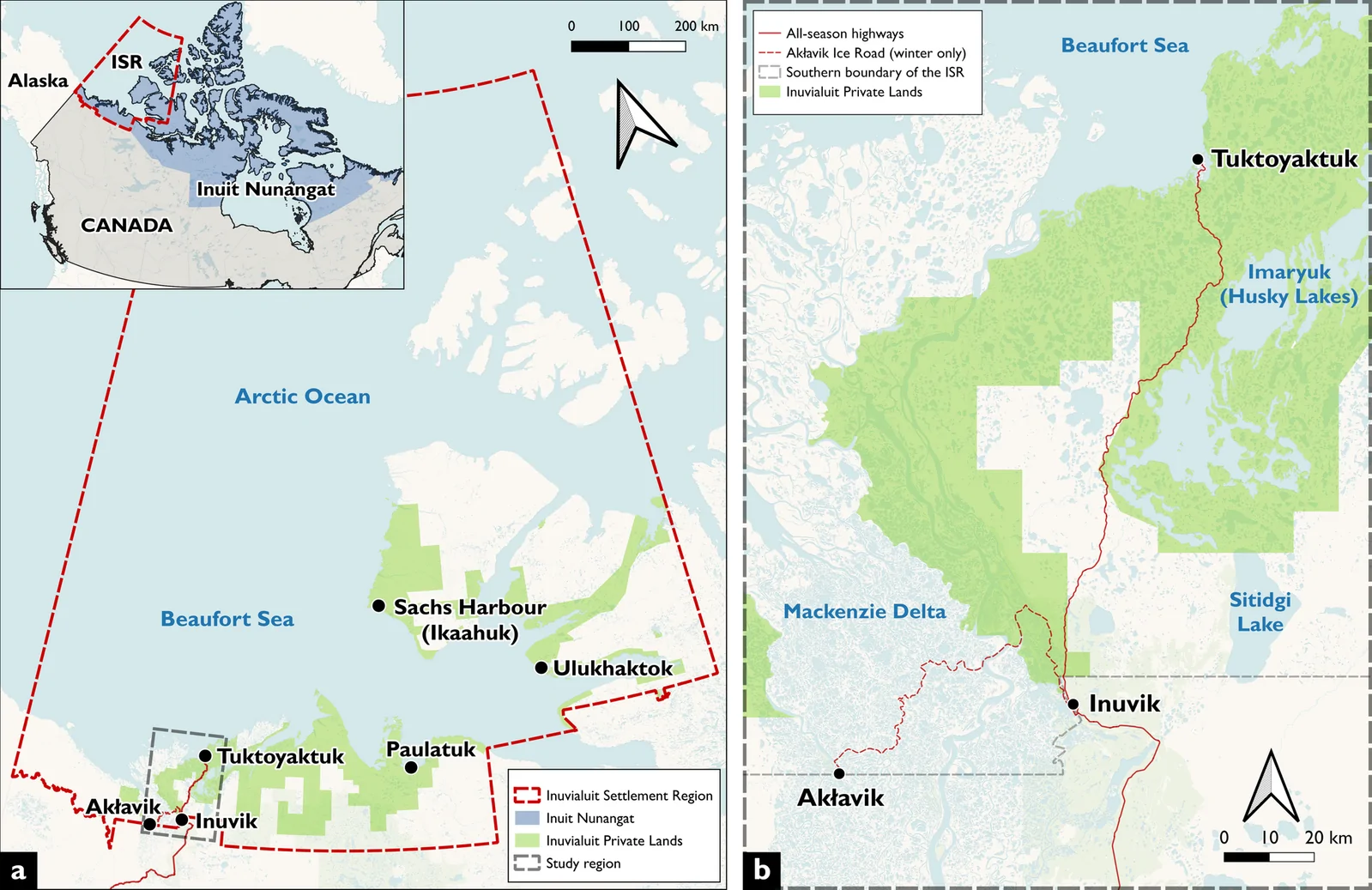
(**a**) Map showing the Inuvialuit Settlement Region within Inuit Nunangat and Canada. (**b**) Map of the study region. Maps created with QGIS 4.0. Map tiles by CartoDB (CC BY 3.0). Data by OpenStreetMap (ODbL) and Statistics Canada (Statistics Canada Open Licence)

The impact of beavers on harvesting activities emerged as a major concern for communities in the Inuvialuit Settlement Region (ISR) by 2017. The ISR, located in north-west Canada, consists of the traditional lands and sea of the Inuvialuit and forms the western part of Inuit Nunangat (Fig. 1a). Inuvialuit hunters reported seeing beaver dams and lodges west of the Mackenzie River in 2008 and 2009 (Jung et al., 2016). By 2017, three communities in the south-western portion of the ISR were raising concerns about beaver population change: Inuvik, Tuktoyaktuk, and Aklavik. By 2023, people from the coastal community of Paulatuk were also reporting beaver sightings on social media.

These community concerns were reported through local Hunters and Trappers Committees (HTCs) to the Inuvialuit Game Council (IGC), a co-management body appointed by HTCs that represents collective Inuvialuit interests in wildlife management in the ISR (IFA, 2005). These concerns focused on the impact of beaver dams on key fishing sites, as well as the potential for beavers to affect the ecosystem of the Imaryuk (or Husky Lakes): a chain of saltwater lakes south of the Tuktoyaktuk Peninsula that forms an important source of fish for many Inuvialuit. In 2017 and 2018 the IGC responded to the issue by introducing a trial sample programme, organised with the Department of Environment and Natural Resources (ENR, now Environment and Climate Change Canada), that offered payments to trappers in exchange for beaver samples (skull, castor sacs, or baculum). A payment of $50 CAD (Canadian dollars) in 2017 was increased to $100 CAD in 2018 to encourage trappers, but the programme ended early amid increasing costs and Inuvialuit concerns about wastage of beaver meat (Scott, 2018; Malbeuf, 2018).

Our research focuses on the impact of beavers on Inuvialuit livelihoods and wellbeing, as part of the interdisciplinary and collaborative BARIN project (*Beavers* *and Socio-Ecological* *Resilience in* *Inuit* *Nunangat*). It examines how the biophysical consequences of beaver population change affect Inuvialuit communities. We conducted qualitative research to address two major questions: (1) *how does beaver population change in the ISR impact Inuvialuit livelihoods*,* especially in relation to harvesting activities?* and (2) *how does the impact of beavers on Inuvialuit livelihoods affect Inuvialuit wellbeing?* In addressing this second question we focus on ‘evaluative wellbeing,’ referring to participants’ open assessments of positive criteria in their own lives (MacKerron, 2012: 706).

## Study Region

The Inuvialuit are the Inuit of the western Canadian Arctic, with close connections to Alaskan Iñupiat. The traditional lands, sea and ice of the Inuvialuit constitute the Inuvialuit Settlement Region (ISR), around the lower Mackenzie Delta and on the shores of the Beaufort Sea (Fig. 1a). The ISR extends across parts of the Yukon and Northwest Territories in Canada and contains six communities, all located in the Northwest Territories: Inuvik/Inuuvik (population in 2021: 3,137), Tuktoyaktuk/Tuktuyaaqtuuq (937), Aklavik/Akłarvik (536), Uluhaktok/Uluqsaqtuuq (408), Paulatuk/Paulatuuq (298) and Sachs Harbour/Ikaahuk (104) (Statistics Canada, 2022a). In total there were 3,145 people registered as Inuit/Inuvialuit in the ISR in 2021, of whom 1,260 lived in Inuvik (40% of the town’s total population), 815 in Tuktoyaktuk (87%), and 320 in Aklavik (60%) (Statistics Canada, 2022b). Inuvik and Aklavik fall within both the ISR and the neighbouring Gwich’in Settlement Area, and both communities have significant Gwich’in populations.

The ISR was legally defined in the 1984 Inuvialuit Final Agreement (IFA) between the Inuvialuit and the Government of Canada. This agreement recognised Inuvialuit traditional use and occupancy of their traditional lands, enshrining Inuvialuit harvesting rights and ensuring Inuvialuit participation in land and wildlife management through the establishment of co-management bodies such as the IGC and the HTCs (IFA, 2005).

Inuvialuit engage in subsistence harvesting activities such as hunting, trapping, fishing, whaling, and berry-picking. Inuvialuit also participate in fur trapping for commercial purposes. Harvesting activities vary by season: Inuvialuit fish, hunt caribou, and trap all year round, hunt geese and grizzly bears in the spring, beluga whales in summer, and seals and polar bears in the winter (ICC, TCC and ACC, 2006). Inuvialuit mostly fish for coney, loche/burbot, trout, whitefish, Arctic grayling, Arctic char and Arctic cisco. Inuvialuit trapping focuses on lynx, muskrat, wolf, wolverine and fox. Very few Inuvialuit now actively trap beaver.

Although fewer Inuvialuit hunt, trap or fish full time today, these activities remain central to Inuvialuit identity. Harvesting is strongly associated with cooperative activity and with periods of sociality on the land, when families or communities work together to harvest traditional food. Elders and others unable to directly participate in harvesting are assisted by food-sharing initiatives organised by HTCs and the Inuvialuit Community Economic Development Organisation (ICEDO), which pay active harvesters for food that is redistributed among Inuvialuit communities (Brown, 2024).

Our research focuses on the communities of Inuvik, Tuktoyaktuk, and Aklavik in the southwestern portion of the ISR, around the lower Mackenzie Delta (Fig. 1b). This is an ecological transition zone between the boreal forest to the south and the low Arctic tundra found further north. The presence of the Mackenzie River extends the treeline north, though trees along the river tend to be stunted by permafrost. Low-lying shrubs such as willows and birch are common on the tundra and are used by beavers as food and building materials in the absence of trees.

## Methods

The present study addresses Inuvialuit research priorities, following Priority Area 3 of the National Inuit Strategy on Research (ITK, 2018). The research was developed in collaboration with the Joint Secretariat of the ISR and the Fisheries Joint Management Committee (FJMC), while key project objectives were determined through face-to-face and online discussions with members of the Inuvialuit Game Council (IGC), the Inuvialuit Regional Corporation (IRC), and the Inuvik, Tuktoyaktuk, and Aklavik HTCs.

We conducted semi-structured interviews and participatory mapping with 25 Inuvialuit knowledge holders (17 men and 8 women) from Inuvik (6 men, 1 woman), Tuktoyaktuk (5 men, 2 women) and Aklavik (6 men, 5 women), which were mainly conducted between July and September 2023 (Inuvik and Tuktoyaktuk) and in February 2024 (Inuvik and Aklavik). All participants were or (in the case of some elders, had been) active harvesters, and were recognised by HTCs as holding specialised knowledge about life on the land and Inuvialuit harvesting activities. The gender bias among participants may have led to an emphasis in our results on traditionally male harvesting activities such as hunting and trapping, with less attention paid to traditionally female activities such as berry-picking and food processing.

Four participants were also members of the Imaryuk Monitoring Program, an Indigenous guardianship initiative focused on the Imaryuk lake system. Imaryuk Monitors patrol the Inuvik-Tuktoyaktuk Highway (ITH), an all-season access road opened in 2017, monitoring fishing activity in remote lakes that have become accessible from this new road. Imaryuk Monitors also carry out environmental monitoring on fish stocks and fish habitat, often in partnership with outside researchers (FJMC, 2019).

Potential research participants were identified by the HTCs for the three communities, which acted as gatekeeper organisations for the research. This role was specified in our research agreement with Inuvialuit organisations and aligns with the stated purpose of HTCs in the IFA to promote Inuvialuit involvement in research on wildlife in the ISR (IFA, 2005). Recruitment and interviews were conducted in English, as the Indigenous Inuvialuktun languages are spoken by less than 3% of people in the three communities with whom we worked (IRC, 2011). Interviewees were largely self-selected, with the sample limited to those who expressed interest in participation through the HTCs. We paid research participants honoraria of $125 CAD per research session.

Interviews drew on an interview guide consisting of a flexible list of open-ended questions grouped into four main themes: livelihood (background, involvement in harvesting); observations of environmental change; observations of beaver population change and impacts of beavers on harvesting; and finally, wellbeing (factors contributing to wellbeing, the impact of beavers on wellbeing). As the term ‘wellbeing’ had little resonance for most participants, the interview guide framed questions on this topic in terms of ‘what makes life good,’ or ‘what is needed for a good life.’ While every interview covered these four main themes, the order and specific questions asked varied. Interviews relied heavily on follow-up questions adapted to the topics and concerns raised by individual participants, following the norms of semi-structured interview practice (Brinkmann & Kvale, 2018) and guidance on interviewing Inuit elders (Owlijoot, 2008). Maps were used to aid interviews and to contribute to an online story map, created with ArcGIS Online, presenting selected quotes from interviews in a publicly accessible format.

We obtained informed consent from research participants using written consent forms where possible or recording consent using a pre-prepared script. Research participants had the option to conduct interviews without the use of an audio recorder (an option chosen by 4 participants), for which the interviewer relied primarily on improvised shorthand with verbatim note-taking used for key comments. We transcribed all audio-recorded interviews with the aid of a local instance of the Whisper V3 speech recognition software, manually correcting automatically generated transcripts and coding these thematically using NVivo© 14.23.3 to aid the retrieval of key quotes. Coding focused on beaver impacts (distinguishing between observed impacts and concerns), knowledge about beaver population change, knowledge of other environmental changes, and factors contributing to wellbeing. Interviews were analysed using a reflexive thematic approach (Braun & Clarke, 2022) drawing on immersion in the material through field research and the correction of transcripts. This article draws on both interview recordings and notes from unrecorded interviews, but all block quotes are taken from transcriptions of recordings.

At the end of the research project, all interview recordings, interview transcripts, and notes on unrecorded interviews were passed on to the Joint Secretariat, acting on behalf of the IGC, to contribute to an archive of Inuvialuit knowledge. This is intended to ensure that Inuvialuit organisations maintain ownership of and access to research material gathered with Inuvialuit research participants, following Priority Area 4 of the National Inuit Strategy on Research (ITK, 2018). Material held in this archive will only be accessible with the permission of the IGC.

The IGC made it clear early on that it was important for them to know whose knowledge was being stored within this archive, meaning that the names of specific interviewees needed to remain attached to interview recordings and transcripts. This meant that we could not offer research participants full anonymity. This follows the understanding that Indigenous knowledge is bound up with the expertise and lived experience of the individuals who hold it (Cruikshank, 2004), with anonymisation effectively severing the experiential and relational connections on which such knowledge depends. However, we did offer participants the option to remain confidential in published outputs (an option chosen by only 1 participant, anonymised here). The decision not to offer full anonymity followed our commitment to advance Inuvialuit decision-making in research, to use Inuvialuit-specific ethical research guidelines, and to support Inuvialuit self-determination in research, following Priority Areas 1, 2 and 4 of the National Inuit Strategy on Research (ITK, 2018).

## Results

### Beaver Population Change

All participants described noticing a major increase in the numbers of beavers, with beavers now ‘*everywhere*,* in every river*,* in every lake*,* in every channel*’ (Arey, 2023). Eleven participants stated that beavers were not a new species in the lower Mackenzie Delta (Arey, 2023; Arey D, 2024; Emaghok, 2023; Gordon D Jr., 2024; Gray, 2023; Gruben R, 2023; Gruben S Sr., 2023; Gruben, 2024; Klengenberg, 2024; Pokiak, 2024; Wade, 2023), but described them becoming much more common and as ‘*progressing farther north*’ (Wade, 2023). Participants described seeing shifts in beaver distribution patterns: ‘*you’re starting to see them on the rivers making houses as well*,* so… not just in the creeks or the lakes*’ (Storr, 2024). Research participants in Tuktoyaktuk described beavers ‘*swimming in the harbour*’ (Elias, 2023; Emaghok, 2023), something that was also mentioned by participants in Inuvik and Aklavik (Dillon M Sr., 2023; Arey D, 2024; Arey R, 2024). Five participants described seeing areas where beavers had stripped out the vegetation along creeks and around lakes (Dillon M Jr., 2023; Gordon P, 2024; McLeod M, 2024; Pokiak, 2024; Storr, 2024). Estimates for when people first started noticing the increase in beavers varied by community. In Aklavik, four participants described the issue becoming more apparent over the past ten years (Arey D, 2024; Gruben, 2024; McLeod B, 2024; McLeod M, 2024). Elder Annie B. Gordon described the beaver population increasing since the ‘*early seventies*,* eighties*,* maybe*,’ but that now ‘*every place you travel you seem to see beavers*’ (Gordon AB, 2024). This was echoed by J.D. Storr, a younger harvester who sits on the IGC, who described beaver numbers increasing steadily over decades but becoming more visible in the past few years (Storr, 2024). Three participants described beaver numbers reaching a peak a few years ago before declining after a large beaver hunt two years ago (Arey R, 2024; Gordon D Jr., 2024; McLeod M, 2024), with two suggesting that a recent decline in beaver numbers might reflect a natural cycle like those seen in rabbit and muskrat populations (Arey R, 2024; McLeod M, 2024).

In Inuvik, Max Kotokak Sr., a senior Imaryuk Monitor originally from Tuktoyaktuk, described beavers becoming more apparent ‘*about four or five years ago*,’ and connected this increase in visibility to the opening of the ITH in 2017: ‘*before the road*,* we didn’t really know*,* because*,* you know*,* like I said*,* never travelled around. Never travelled around during the summer*’ (Kotokak, 2023). Similarly, Camellia Gray described noticing more beavers at some point ‘*over the past five years*’ (Gray, 2023). Brian Wade, director of ICEDO and a former president of the Inuvik HTC, noted that a ‘*big*,* big increase*’ became particularly apparent in ‘*2019*,* 2020*,’ at which point co-management bodies started to pay more attention to the issue (Wade, 2023).

In Tuktokyaktuk, Sammy Gruben Sr. similarly described beavers becoming more apparent ‘*four or five years ago*’ (Gruben S Sr., 2023). Others gave earlier estimates. Jim Elias, who represents Tuktoyaktuk on the IGC and whose trapping area extends far to the south and east of the settlement, described noticing a beaver distribution change some decades before it became apparent in Tuktoyaktuk itself: ‘*they started coming up this way probably in the 80s*,* and then now it’s more noticeable than that. Late 80s and 90s*,* you started having beaver houses all over the place*’ (Elias, 2023). Lennie Emaghok, a senior Imaryuk Monitor, started noticing an increase in beaver lodges in areas used for geese hunting in the spring ‘*probably 10*,* 12 years ago*’ (Emaghok, 2023).

Explanations given by participants for the increase in the number of beavers included the warming climate and longer summers (Gordon P, 2024; Gray, 2023; Gruben, 2024; Wade, 2023) and the impact of forest fires and habitat destruction further south (Elias, 2023; Gordon P, 2024; Gruben R, 2023; Gruben S Sr., 2023). Several participants linked beaver population change to observations of increased shrub growth (Emaghok, 2023; Gray, 2023; Wade, 2023):

*Back in the 80s*,* we didn’t see very many beaver houses and stuff around the country […] we thought they’d have starved themselves because they couldn’t eat nothing […] because there’s not enough willows to chew on and stuff like that. But now*,* today*,* you could see a sprout of a willow and go back there five years*,* it’s like three feet tall*,* three*,* four feet tall. It’s so much warmer weather. Those willows are growing a hundred times faster than when they used to grow. […] There’s places where we never had willows that there’s willows.* (Elias, 2023)

A major factor for many participants was the decline in commercial fur trapping (Arey, 2023; Arey D, 2024; Arey R, 2024; Gordon D Jr., 2024; Gruben R, 2023; McLeod B, 2024; McLeod M, 2024; Storr, 2024; Wade, 2023). Like many research participants, Patrick Gordon, a full-time harvester in Aklavik, drew a direct connection between beaver population change and the removal of this limiting factor:

*there must be just not enough harvesting [beavers]*,* not enough people*,* you know*,* maintaining them. Like*,* you know*,* they say*,* no*,* no one needs to really hunt them. And then they just start overpopulating…* (Gordon P, 2024).

Many participants also described beavers as having been introduced to or ‘implanted’ in the lower Mackenzie Delta by the Government of Canada at some point in the mid-twentieth century (Arey, 2023; Arey D, 2024; Arey R, 2024; Dillon M Sr., 2023; Elias, 2023; Gordon AB, 2024; Kotokak, 2023; Storr, 2024).

### Beaver Impacts on Fishing

The impact of beavers on fish populations emerged as the primary concern for almost all research participants in all three communities. Dean Arey, chair of the IGC and an active harvester in Aklavik, described beavers as a ‘pest’ that disrupt key fishing sites:

*And now all our fishing creeks are buggered up from the beavers because they’re making dams. And it’s like*,* we had a lot of good fishing creeks where we used to go jiggle in the fall. And now they’re all getting no good because of the beaver dams. […] They’re overpopulated. They’re just like a pest now.* (Arey D, 2024)

Participants focused on the way beaver dams lower the water level in lakes (Teddy, 2023) and key fishing creeks, and blocking the passage of the minnows on which larger fish feed:

*Culturally*,* what we do in the fall time when the ice first goes on is we find a creek. It doesn’t matter the size. It’s six feet across or two feet across. As long as it’s flowing and has minnows that are coming out. And then we fish at the bottom of that creek and that’s where we catch our fish. A lot of the good fishing creeks are dammed up now. So*,* if they’re not flowing*,* there’s no minnows*,* which means that there’s no fish.* (Wade, 2023)

Several participants described observing first-hand the effect of beavers on the water level and availability of fish in specific creeks, particularly around Aklavik:

*There’s a creek in Taylor Channel*,* it’s Joe Arey’s Creek. That one’s almost dried right out*,* Martin’s [Creek]*,* the one I was in*,* it’s really shallow now*,* and you need to be able to drive in all these creeks right to the lake*,* and now you have a tough time making it in the little ways*,* because the water is really shallow. […] Jackfish [Creek]*,* same thing. Jim Firth’s [Creek]. Almost all of them are kind of affected by beavers in the creek. They’re damming up the creeks and making it a lot harder for fish*,* people are catching a lot less fish than what we usually catch.* (Storr, 2024)

Similarly, senior Imaryuk Monitor Lennie Emaghok described seeing the impact of beaver dams on Hans Creek and Gungi Creek, both south of Tuktoyaktuk:

*Two years ago*,* at Hans Creek […] it was really low water*,* similar to what it is today*,* really low water*,* like hardly anything running in Hans Creek. So*,* we actually found out there’s a beaver dam […] When the beavers first plugged up the Gungi Creek near the arch bridge*,* when we were taking the beaver dam apart*,* you could actually see the fish stuck in the willows. […] We actually photographed a few fish that got actually stuck in between the willows and died in there.* (Emaghok, 2023)

### Beaver Impacts on Drinking Water

A second major concern described by most participants in all three communities related to the impact of beavers on drinking water. Seventeen participants described concerns about the risk of ‘beaver fever,’ a term used to refer to waterborne infections such as giardiasis (Arey, 2023; Arey D, 2024; Arey R, 2024; Dillon M Jr., 2023; Dillon M Sr., 2023; Elias, 2023; Gordon AB, 2024; Gordon D, 2024; Gordon P, 2024; Gruben C, 2023; Gruben R, 2023; Gruben S Sr., 2023; Gruben, 2024; Kotokak, 2023; Pokiak, 2024; Storr, 2024; Wade, 2023). Miles Dillon Sr., who lives in Inuvik and works in season as a wildlife monitor for visiting researchers, described concerns with the cleanliness of water where beavers have been:

*More beavers is dangerous for the water because of beaver fever*,* how you say that word… Giardia. So*,* there’s a big concern for our consumption of water. We don’t want to get sick. […] And where [beavers] wash them[selves]? You never want to go around that. Yeah. It’s just like oil and gas*,* so many parts per million. You’re gone. […] God*,* that’s really nasty stuff. You can smell [the water] from a long ways.* (Dillon M Sr., 2023)

These concerns have driven behavioural changes, with people avoiding lake or creek water when out on the land and instead relying on bottled water bought in town:

*I had an elder that passed away just a year ago. He told me when he was getting sick*,* he told me*,* don’t drink water from the lake and the rivers. The creeks. There’s too much beavers. There’s lots of otters. And muskrats. And marmots […] He said*,* the water is not safe from there. But they piss*,* you know*,* in the water. The water is not safe anymore. And I don’t drink water. Now when we go travelling*,* we have to get trucks of water to go out. Anywhere we travel*,* we have to try to get us water to take along with us.* (Gordon AB, 2024)

### Beaver Impacts on Travel

Seven participants in Inuvik and Aklavik spoke of how beaver dams have lowered the water in channels used for summer travel, making traditional routes impassable and forcing people to take longer diversions (Dillon M Sr., 2023; Gordon P, 2024; Gruben, 2024; McLeod B, 2024; Meyook, 2024; Storr, 2024). This is a particular issue for smaller waterways around Aklavik:

*I mean*,* beavers are taking a big toll on a lot of our heritage trails. […] I used those routes*,* like*,* growing up. But my children and their children are not going to use these routes*,* and that’s due to… a lot of it is due to beavers plugging up the main channels to get the water into there.* (Arey, 2023)

Longer routes require more time and fuel, making travel more expensive, while detours through the ocean may be more dangerous. Kevin Arey, an Imaryuk Monitor originally from Aklavik, noted that it is not always possible to distinguish whether low water levels are caused by beaver dams or by slumping from permafrost thaw (Arey, 2023). Three participants suggested that beavers might be contributing to slumping (Dillon M Jr., 2023; Elias, 2023; Gruben R, 2023), but the issue of low water levels emerged as a broader issue that could not be attributed to one cause (Arey D, 2024; Wade, 2023).

Beavers can also make travel in winter riskier: the higher water temperature around beaver lodges creates hidden patches of thin ice that pose a risk for travel by snowmobile (Elias, 2023; Gordon D, 2024; Wade, 2023), while the flooding caused by beaver dams can lead to overflow that creates areas of slush (Arey R, 2024; Elias, 2023; Emaghok, 2023; McLeod B, 2024; Wade, 2023). Similarly, a burst beaver dam can create dangerous air pockets under the surface of the ice:

*Beavers always close off creeks […] by the time winter comes and the ice*,* it gets hard*,* and the dam breaks and you get that much air between the ice and the water. […] It’s hanging ice*,* they call it. And once you go on*,* it will drop. […] it’s like you can’t see it. It’s like an air bubble underneath the ice after that water escapes. […] And the air pockets all the way across the lake and you don’t know. Plus the snow*,* you can’t see no cracks. […] So*,* it’s kind of dangerous to see a beaver dam. You’re second-guessing yourself.* (Gordon D, 2024)

### Beaver Impacts on other Mammals

Few participants described beavers impacting the distribution of other mammals, but some linked beavers to an increase in otters (Elias, 2023; Kotokak, 2023). Three participants also raised the concern that otters might affect fish numbers (Arey, 2023; Gruben S Sr., 2023; Storr, 2024). Four participants noted the way muskrat live alongside beavers and benefit from their dams, but described recent increases in muskrat as following a natural cycle (Dillon M Sr., 2023; Elias, 2023; Gordon P, 2024; Wade, 2023).

Jim Elias commented on how beaver engineering has led to more open surface water around Tuktoyaktuk, increasing humidity and bringing in fog off the land. He also drew a link between these environmental changes and an increase in moose:

*And I think because of the beavers*,* that’s why we’re getting more moose coming farther north. Because when a beaver changes an area*,* we’ve got so flat land up here. If a beaver put a dam in a creek or mouth of a river*,* that lake gets wider and stuff and then you have a marsh. And then we all know moose like to eat plants that are in that water.* (Elias, 2023)

### Factors Contributing to Wellbeing

Knowledge holders in all three communities consistently identified several common factors as contributing to a sense of wellbeing: the ability to go out harvesting on the land; access to traditional food, especially caribou, *maktaaq* (whale skin and blubber), fish, and berries; the importance of family and community; and the importance of sharing knowledge with future generations. Michelle Gruben, who works as Resource Person for the Aklavik HTC, described some of the interconnections between these factors:

*It’s just… to get out on the land*,* to breathe that fresh air*,* leave this crazy cell phone home*,* and just reconnect to the land. Because the land is so powerful. […] I think you just need to get on the land to replenish your mind*,* your mind*,* body and soul. And share this kind of knowledge with… so it could be around for years to come. You know*,* so we could learn from each other.* (Gruben, 2024)

These factors align with those identified in the literature on wellbeing in the ISR and other parts of Inuit Nunangat, which have consistently emphasised the centrality to Inuit wellbeing of collective identity (Kral & Idlout, 2024), subsistence harvesting (Bunce et al., 2016; Snook et al., 2022; Turner et al., 2018), and the therapeutic significance of being on the land (Robertson & Ljubicic, 2019). Participants described the experience of going out on the land as ‘*beautiful*’ (Gruben C, 2023), as ‘*unbelievable*’ (Kotokak, 2023), and spoke of how ‘*having that connection to the land is what makes life good […] it recentres me and refocuses me*’ (Wade, 2023). Some also mentioned the practical benefits of living off the land. Camellia Gray explained that, ‘*it’s too expensive to live off the store. And it’s more healthier to live off our traditional food. Lots of protein*,* vitamins*,* what we need’* (Gray, 2023). Similarly, Annie B. Gordon linked increases in health problems among younger people with a move away from traditional food:

*Because they’re living on fast foods. Some of them don’t even touch caribou meat. […] That’s why we have lots of different sicknesses now. We never used to see people get sick like that. And when you start eating those kind of food*,* you don’t feel well.* (Gordon AB, 2024)

Fifteen participants identified access to money and the ability to cope with the increased cost of living as a significant factor contributing to their sense of wellbeing (Arey, 2023; Arey D, 2024; Arey R, 2024; Dillon M Sr., 2023; Elias, 2023; Emaghok, 2023; Gordon D Jr., 2024; Gordon P, 2024; Gruben C, 2023; Gruben S Sr., 2023; Gruben, 2024; Gray, 2023; Kotokak, 2023; McLeod B, 2024; Storr, 2024). The high costs of fuel, food and equipment came up in almost all interviews, and repeatedly emerged as the major factor in discouraging people from going out on the land:

*Even our elders used to say that over the years you are going see the changes*,* how we live. And it’s so true. They even said our children*,* our children*,* you got to keep training them to be out on the land. Because this is something that our elders don’t want us to lose. But it’s hard to*,* because the gas price is up*,* everything is up now. Not like long before. I used to go with dog teams and whatnot*,* gas price is up*,* it’s expensive.* (Arey R, 2024)

While all participants identified beavers as a problem, few described them as directly impacting key wellbeing factors such as the ability to go out on the land or obtain traditional food. Instead, when confronted with beaver impacts on fishing creeks, disrupted travel routes or problems with drinking water, participants spoke of the need to ‘*adapt*’ (Arey D, 2024; Gordon P, 2024; Gruben R, 2023; Gruben, 2024; Klengenberg, 2024; McLeod B, 2024; McLeod M, 2024; Storr, 2024; Wade, 2023), to ‘*evolve with the changes*’ (Arey, 2023), to try to ‘*carry on*,* evolve and keep up to the changes*’ (Dillon M Sr., 2023) or to ‘*go with whatever the changes are*’ (Arey R, 2024). Participants spoke of adapting to beavers by finding new fishing creeks and new routes, but also by actively reducing beaver numbers through harvesting incentive programmes (McLeod B, 2024) or by culling beavers near settlements (Arey, 2023).

These participants emphasised the centrality of adaptation to Inuvialuit harvesting: ‘*[We] just got to adapt to whatever’s coming*,* that’s our nature*. […] *We have to adapt to these situations*,* as hunters. Adaptation is key*’ (Gruben R, 2023). This emphasis may reflect the needs of life in a highly dynamic and already changeable environment, and has been linked to a deep-seated ‘valuation of volatility’ among Inuvialuit and Gwich’in (Krause, 2023: 169). As Dean Arey put it,

*You have to change what you’re doing. You have to watch what you’re doing. You have to adapt to the weather. You have to adapt to other things. So as animals*,* they have to adapt to changing warmer waters*,* colder waters*,* all that. They’ve got to adapt*,* too. We’ve got to adapt. They’ve got to adapt. Right?* (Arey D, 2024)

## Discussion

The reflections of Inuvialuit knowledge holders demonstrate the cascading socio-ecological impacts of biophysical processes: environmental and climactic changes in the ISR are driving an ecosystem regime shift with effects that aggravate existing social and environmental challenges for Inuvialuit, increasing pressure on harvesting activities that are already difficult and expensive to maintain.

The issues around beaver population change described by participants covered both observed impacts (dammed-up fishing creeks, diverted routes) and concerns for the future (especially around the longer-term availability of fish and the cleanliness of drinking water). While direct awareness of the scope of beaver impacts varied between participants, the concerns they described remained highly consistent across all three communities and all participants. The three main themes of fishing, drinking water, and travel came up repeatedly. The impact of beavers on waterways used for summer travel was a particular concern in Aklavik, but was also mentioned by participants in Inuvik and Tuktoyaktuk.

As a driver of environmental change, beavers are thoroughly entangled with other environmental and social factors: participants associated beaver expansion with the warming climate, shrubification, and forest fires further south, but also with the history of pre-IFA wildlife management in the ISR and the decline in fur trapping over the latter half of the twentieth century. Similarly, environmental changes linked to beavers – such as lower water levels in creeks in summer and overflow in winter – were also attributed to higher temperatures and the effects of permafrost thaw. Inuvialuit knowledge holders described a complex interrelationship between environmental and social factors with no single driver of change, in which beavers emerge as a visible marker of multiple long-term transformations in the Mackenzie Delta.

Human action plays a key role in this system on a local as well as a global level: while participants described observing the consequences of a warming climate, they also repeatedly emphasised the significance of the reduction in Inuvialuit harvesting activity over the past century and wildlife management policies pursued by Canadian government agencies before 1984. These policies were described by many participants as the key factor in beaver population change, with beavers portrayed as an introduced species. There are parallels here to the history of disputes over muskox management in the Mackenzie Delta (Nagy, 2004; Wishart, 2004), a point raised in interview by J.D. Storr (Storr, 2024). References to beavers being ‘implanted’ in the lower Delta in the mid-twentieth century may be connected to a beaver transplantation conducted by the Canadian Wildlife Service in 1953, in which beavers were taken from areas on the East Branch of the Mackenzie River and released onto five sites around Aklavik (McEwen, 1953). These topics are discussed in greater detail in Pearce and Wheeler (2026).

Participants focused on the impact of beaver engineering on water levels and water quality, with observations of lower water levels in creeks and a greater frequency of areas of stagnant standing water driving most concerns. These concerns are already leading to changes in behaviour, with Inuvialuit travelling further to find new fishing sites, taking long diversions while travelling (involving greater expenditure on fuel), and relying on bottled water instead of creek or lake water while out on the land. These shifts are consistent with changes in harvesting practices described by other researchers working in the Mackenzie Delta (Berkes & Jolly, 2002; Communities of Aklavik, Inuvik, Holman Island, Paulatuk & Tuktoyaktuk et al., 2005; Pearce et al., 2008; Ziegler et al., 2024) and elsewhere in Inuit Nunangat and Arctic and sub-Arctic Alaska (Brinkman et al., 2016; Ford et al., 2007; Wilson et al., 2014).

These changes in behaviour, driven by concerns about beavers, are increasing the financial costs associated with harvesting and exacerbating existing issues around the cost of fuel, food, and equipment. The cost of living emerged as a major wellbeing factor for almost all participants, with increased costs associated with harvesting representing the clearest tangible impact from beavers on Inuvialuit wellbeing. As harvesting becomes more expensive, higher costs limit the ability of Inuvialuit to respond to environmental pressures (especially extreme weather and changes in animal abundance). This constrains Inuvialuit adaptive capacity and increases the exposure of active harvesters to climatic risk. The groups most affected by these changes are those with limited access to economic resources and younger people with limited skills (Andrachuk & Smit, 2012; Ford et al., 2007; Pearce et al., 2009).

Initially, this study aimed to identify whether greater impacts from beavers on wellbeing were associated with demographic characteristics (e.g. age, gender, livelihood). In most cases, however, research participants focused on concerns for the future rather than direct impacts, and described beavers as affecting Inuvialuit on a collective rather than individual level: participants discussed whether future generations would be able to use the same routes or the same fishing sites, how the changing environment would affect the continuity of Inuvialuit knowledge, and whether their community as a whole would be affected by reduced access to clean drinking water. This echoes points made by other researchers working with Inuit peoples on the importance of collective identity for wellbeing (Kral & Idlout, 2024), the collective ownership of knowledge and cultural heritage (Nagy, 2011), and the resilient association between harvesting and collectivity (Stuckenberger, 2006). Beavers, we suggest, are a collective rather than individual concern specifically because of their impact on harvesting activities, which are bound up with intergenerational knowledge transfer, periods of sociality on the land, and food sharing that benefits the whole community. This emphasis on collectivity is further indicated in the consistent use of the first-person plural (*we*, *our*) by participants: while participants described individual observations of the presence of beavers, when discussing impacts on harvesting they spoke of ‘our fishing creeks,’ ‘our traditional food,’ ‘our water,’ ‘our heritage trails,’ etc.

The emphasis participants placed on adaptation when discussing the impacts of beavers on harvesting activities complicates any discussion of the implications for wellbeing. Participants largely rejected the idea that beavers were directly affecting the availability of traditional food or engagement in harvesting. Participants did describe beaver engineering as contributing to making harvesting more difficult, expensive and time-consuming, but stressed the need to adapt to these impacts through changing harvesting patterns and beaver harvesting incentive programmes. The adaptive responses described by participants largely represent reactive strategies involving longer travelling times, higher costs, and trade-offs between harvesting activities. Researchers have commented before on Inuvialuit adaptability in the face of environmental change (Andrachuk & Pearce, 2010; Andrachuk & Smit, 2012; Berkes & Jolly, 2002) and have identified the high cost of fuel and other supplies as a key constraint to adaptation (Pearce et al., 2009). While participants also described beaver population change as contributing to increasing environmental unpredictability, a recurring theme in the literature on this topic (Ayeb-Karlsson et al., 2024; Jolly et al., 2002), most did not identify this as directly detrimental to wellbeing. This could suggest that beavers pose a problem primarily because they interact with other factors, especially the cost of living, that constrain adaptive strategies (see Prno et al., 2011).

This alone does not explain why beavers are such a major issue for Inuvialuit communities. The way beavers transform the environment (redirecting creeks, changing the movement of fish, driving changes in the distribution of other species, even affecting the frequency of inland fog) may have a qualitative effect on the way Inuvialuit experience a connection to the land through harvesting activity. Researchers have commented on the importance of relationality for Inuit communities, emphasising the significance of connections between humans and non-humans established through intergenerational knowledge transfer and reinforced through collective participation in time spent on the land (Robertson & Ljubicic, 2019). Such interrelations have a particular significance for Inuvialuit identity (Lyons, 2010). These points align with comments made by research participants, quoted above.

Beavers make the environment unfamiliar, and in doing so they pose a challenge to these relations: by driving Inuvialuit away from established travelling routes and fishing creeks, and by making drinking water unreliable, their engineering makes it harder to maintain the connections to the land that underpin Inuit identity (Cunsolo Willox et al., 2012; Middleton et al., 2020). While beavers may not directly prevent people from continuing to hunt or fish, they make harvesting more difficult and threaten to disrupt the continuity of knowledge on which Inuvialuit collective identity relies.

## Conclusion

Many Indigenous communities are experiencing major interlinked environmental and social transformations in the face of climatic, environmental, and societal drivers of ecosystem change. Local impacts of both direct climate and environmental changes have been reported by Indigenous people across a wide range of livelihoods, including agriculture, agropastoralism, pastoralism, hunting and harvesting (Reyes-Garcia et al., 2024). Indigenous knowledge and practices, biocultural approaches, and community-based adaptation are all important contributors to resilience; but the rapidity of change, policy and implementation barriers remain ongoing challenges (Davidson-Hunt et al., 2012; Kumar et al., 2025).

The arrival of new species in ecosystems can trigger regime shifts (Shackleton et al., 2018). Similarly, ‘sleeper’ populations can exist at low densities for many years before environmental change triggers substantial and disruptive population increases (Spear et al., 2021). The latter may describe the case of beavers in the Arctic, where ecological changes have generated clear impacts for communities.

These changes have been perceived in a number of ways. In invasive species contexts, critiques towards Western management have addressed the lack of relational approaches that make space for Indigenous responses to regime shifts (Reo et al., 2018; Wehi et al., 2023). There is also recognition, however, of contexts where species sit outside normal relations or are perceived as unnatural (Theodorakea & von Essen, 2016). For Indigenous people the perceived impacts and concerns from environmental change may reflect a combination of these normal and abnormal relations, leading to a focus on both adaptation and Indigenous management systems to guide transition.

For Inuvialuit communities, beavers play a major role in environmental and social transformations driven by climatic, environmental, and social change. Inuvialuit knowledge holders have described how beaver population change in the ISR has become more visible over the past five to ten years, with beavers building dams throughout the lower Mackenzie Delta and extending their range onto the low Arctic tundra around Tuktoyaktuk. As drivers of environmental change beavers are entangled with a range of other social and environmental factors, while their population growth in recent years may reflect social shifts in the ISR as well as the influence of a warming climate.

Major community concerns include the impact of beavers on fishing, on the availability of clean drinking water, and on travelling routes. Inuvialuit concerns focus on collective rather than individual impacts, with the challenges associated with beavers understood in terms of their future potential to affect community health, the continuity of knowledge, and the maintenance of harvesting activity. We suggest that beaver population change has two main impacts on Inuvialuit wellbeing: first, because they exacerbate challenges posed by the high cost of living in the ISR; and second, because their engineering makes the environment unfamiliar, potentially disrupting the continuity of knowledge, connections to the land, and collective identity. Inuvialuit descriptions of beaver impacts on livelihoods show how ecosystem regime change, driven by biophysical and environmental transformations, can have wide-ranging socio-ecological consequences. Ecosystem regime change is not only an ecological issue, but one with major social implications for Indigenous groups.

## Data Availability

No datasets were generated or analysed during the current study.

## References

[CR1] Arey, D. (2024). Interviewed by Callum Pearce. 9 February, Aklavik.

[CR2] Arey, K. (2023). Interviewed by Callum Pearce. 15 August, Inuvik.

[CR3] Arey, R. (2024). Interviewed by Callum Pearce. 15 February, Aklavik.

[CR4] Dillon, M. Jr. (2023). Interviewed by Callum Pearce. 7 August, Inuvik.

[CR5] Dillon, M. Sr. (2023). Interviewed by Callum Pearce. 7 August, Inuvik.

[CR6] Elias, J. (2023). Interviewed by Callum Pearce. 30 July, Tuktoyaktuk.

[CR7] Emaghok, L. (2023). Interviewed by Callum Pearce. 1 August, Tuktoyaktuk.

[CR8] Gordon, A. B. (2024). Interviewed by Callum Pearce. 16 February, Aklavik.

[CR9] Gordon, D. Jr. (2024). Interviewed by Callum Pearce. 13 February, Aklavik.

[CR10] Gordon, P. (2024). Interviewed by Callum Pearce. 14 February, Aklavik.

[CR11] Gray, C. (2023). Interviewed by Callum Pearce. 8 August, Inuvik.

[CR12] Gruben, C. (2023). Interviewed by Callum Pearce. 27 July, Tuktoyaktuk.

[CR13] Gruben, M. (2024). Interviewed by Callum Pearce. 12 February, Aklavik.

[CR14] Gruben, R. (2023). Interviewed by Callum Pearce. 25 July, Tuktoyaktuk.

[CR15] Gruben, S. Sr. (2023). Interviewed by Callum Pearce. 27 July, Tuktoyaktuk.

[CR16] Klengenberg, C. (2024). Interviewed by Callum Pearce. 28 February, Fairbanks, Alaska.

[CR17] Kotokak, M. Sr. (2023). Interviewed by Callum Pearce. 22 July, Inuvik.

[CR18] McLeod, B. (2024). Interviewed by Callum Pearce. 16 February, Aklavik.

[CR19] McLeod, M. (2024). Interviewed by Callum Pearce. 14 February, Aklavik.

[CR20] Meyook, P. (2024). Interviewed by Callum Pearce. 14 February, Aklavik.

[CR21] Pokiak, V. (2024). Interviewed by Callum Pearce by telephone. 21 September, Tuktoyaktuk.

[CR22] Storr, J. D. (2024). Interviewed by Callum Pearce. 12 February, Aklavik.

[CR23] Teddy, J. (2023). Interviewed by Callum Pearce. 26 July, Tuktoyaktuk.

[CR24] Wade, B. (2023). Interviewed by Callum Pearce by MS Teams video call. 7September.

[CR25] Andersen, T., Carstensen, J., Hernández-García, E., & Duarte, C. M. (2009). Ecological thresholds and regime shifts: approaches to identification. *Trends in Ecology and Evolution*, *24*(1), 49–57. 10.1016/j.tree.2008.07.014 18952317

[CR26] Andrachuk, M., & Pearce, T. (2010). Vulnerability and adaptation in two communities in the Inuvialuit Settlement Region. In G. K. Hovelsrud, & B. Smit (Eds.), *Community adaptation and vulnerability in Arctic regions* (pp. 63–81). Springer.

[CR27] Andrachuk, M., & Smit, B. (2012). Community-based vulnerability assessment of Tuktoyaktuk, NWT, Canada to environmental and socio-economic changes. *Regional Environmental Change*, *12*, 867–885. 10.1007/s10113-012-0299-0

[CR28] Ayeb-Karlsson, S., Hoad, A., & Trueba, M. L. (2024). 'My appetite and mind would go’: Inuit perceptions of (im)mobility and wellbeing loss under climate change across Inuit Nunangat in the Canadian Arctic. *Humanities and Social Sciences Communications*, *11*, 227. 10.1057/s41599-024-02706-1

[CR29] Baker, K., Hausner, V. H., Ramsay, J., & Wheeler, H. C. (2025). What evidence exists on the interlinkages between ecological and societal impacts of borealisation of the arctic? A systematic map protocol. *Environmental Evidence*, *14*(1), 15. 10.1186/s13750-025-00367-4 40751171 PMC12317435

[CR30] Berkes, F., & Jolly, D. (2002). Adapting to climate change: social-ecological resilience in a Canadian western Arctic community. *Conservation Ecology*, *5*(2), 18. 10.5751/es-00342-050218

[CR31] Braun, V., & Clarke, V. (2022). *Thematic Analysis: A Practical Guide*. Sage.

[CR32] Brinkman, T. J., Hansen, W. D., Chapin, F. S., Kofinas, G., BurnSilver, S., & Rupp, T. S. (2016). Arctic communities perceive climate impacts on access as a critical challenge to availability of subsistence resources. *Climatic Change*, *139*, 413–427. 10.1007/s10584-016-1819-6

[CR33] Brinkmann, S., & Kvale, S. (2018). *Doing Interviews. *Second edition. Sage.

[CR34] Brown, B. (2024). Food Security for Inuvialuit. Inuktitut Magazine, summer 2024. https://www.itk.ca/food-security-for-inuvialuit/. Accessed 10 Oct 2024

[CR35] Bunce, A., Ford, J., Harper, S., Edge, V., & IHACC Research Team. (2016). Vulnerability and adaptive capacity of Inuit women to climate change: A case study from Iqaluit, Nunavut. *Natural Hazards*, *83*, 1419–1441. 10.1007/s11069-016-2398-6

[CR36] CIHR, NSERC, and SSHRC (Canadian Institutes of Health Research, Natural Sciences and Engineering Research Council of Canada, and Social Sciences and Humanities Research Council of Canada). (2022). *Tri-Council Policy Statement: Ethical Conduct for Research Involving Humans (TCPS 2)*. Government of Canada / Gouvernement du Canada. Accessed 17 September 2024. https://ethics.gc.ca/eng/documents/tcps2-2022-en.pdf

[CR37] Communities of Aklavik, Inuvik, Holman Island, Paulatuk and Tuktoyaktuk, Nickels, S., Buell, M., Furgal, C., & Moquin, H. (2005). *Unikkaaqatigiit: Putting the Human Face on Climate Change; Perspectives from the Inuvialuit Settlement Region*. Joint publication of Inuit Tapiriit Kanatami, Nasivvik Centre for Inuit Health and Changing Environments at Université Laval, and the Ajunnginiq Centre at the National Aboriginal Health Organization.

[CR38] Cruikshank, J. (2004). Uses and Abuses of ‘Traditional Knowledge’: Perspectives from the Yukon Territory. In D. G. Anderson, & M. Nuttall (Eds.), *Cultivating Arctic Landscapes: Knowing and Managing Animals in the Circumpolar North* (pp. 17–32). Berghahn.

[CR39] Cunsolo Willox, A., Harper, S. L., Ford, J. D., Landman, K., Houle, K., Edge, V. L., & the Rigolet Inuit Community Government. (2012). From this place and of this place: Climate change, sense of place, and health in Nunatsiavut, Canada. *Social Science and Medicine*, *75*, 538–547. 10.1016/j.socscimed.2012.03.043 22595069

[CR40] Davidson-Hunt, I. J., Turner, K. L., Mead, A. T. P., Cabrera-Lopez, J., Bolton, R., Idrobo, C. J., Miretski, I., Morrison, A., & Robson, J. P. (2012). Biocultural design: a new conceptual framework for sustainable development in rural indigenous and local communities. *SAPIENS: Surveys and Perspectives Integrating Environment and Society,**5*(2). No page numbers. https://journals.openedition.org/sapiens/1382

[CR41] FJMC (Fisheries Joint Management Committee). (2019, 3 April).* Imaryuk*. https://fjmc.ca/imaryuk/. Accessed 28 Mar 2026

[CR42] Folke, C. (2006). Resilience: The emergence of a perspective for social–ecological systems analyses. *Global Environmental Change*, *16*(3), 253–267. 10.1016/j.gloenvcha.2006.04.002

[CR43] Ford, J. D., Pearce, T., Canosa, I. V., & Harper, S. (2021). The rapidly changing Arctic and its societal implications. *Wiley Interdisciplinary Reviews: Climate Change*, *12*(6), e735. 10.1002/wcc.735

[CR44] Ford, J. D., Smit, B., Wandel, J., Allurut, M., Shappa, K., Ittusarjuat, H., & Qrunnut, K. (2007). Climate change in the Arctic: current and future vulnerability in two Inuit communities in Canada. *The Geographic Journal*, *174*(1), 45–62. 10.1111/j.1475-4959.2007.00249.x

[CR45] Fossheim, M., Primicerio, R., Johannesen, E., Ingvaldsen, R. B., Aschan, M. M., & Dolgov, A. V. (2015). Recent warming leads to a rapid borealization of fish communities in the Arctic. *Nature climate change*, *5*(7), 673–677. 10.1038/nclimate2647

[CR46] Glass, T. W., Tape, K. D., Clark, J. A., Johnston, S. E., Jones, B. M., Kehoe, P., Loranty, M. M., Maio, C. V., Rangel, R. C., & Zavoico, V. S. (2025). Ecophysical dynamics of beaver ponds in Arctic Alaska: Implications for permafrost, aquatic habitat, and water quality. *Ecosphere*, *16*(9), e70371. 10.1002/ecs2.70371

[CR47] Hole, G. M., Büntgen, U., Wang, Y., DeVries, B., Rees, G., & Wheeler, H. C. (2026). Dendrochronology and remote sensing reveal beaver occupancy and colonization dynamics in an expanding Arctic population. *Ecosphere*, *17*(3), e70557. 10.1002/ecs2.70557

[CR48] Huntington, H. P., Loring, P. A., Gannon, G., Gearheard, S. F., Gerlach, S. C., & Hamilton, L. C. (2018). Staying in place during times of change in Arctic Alaska: the implications of attachment, alternatives, and buffering. *Regional Environmental Change*, *18*, 489–499. 10.1007/s10113-017-1221-6

[CR49] ICC (Inuuvik Community Corporation) TCC (Tuktuuyaqtuuq Community Corporation), and ACC (Akłarvik Community Corporation). (2006). *Inuvialuit Settlement Region Traditional Knowledge Report*. Calgary: Mackenzie Project Environmental Group.

[CR50] IRC (Inuvialuit Regional Corporation) (2011). Taimani - At That Time: Inuvialuit Timeline Visual Guide. Inuvik: Inuvialuit Regional Corporation. https://irc.inuvialuit.com/documents/taimani-at-that-time-inuvialuit-timeline-visual-guide/. Accessed 2 June 2023

[CR51] IFA (Inuvialuit Final Agreement) as amended (2005). Consolidated version. https://irc.inuvialuit.com/wp-content/uploads/2023/10/Inuvialuit_Final_Agreement_2005.pdf. Accessed 5 Aug 2024

[CR52] ITK (Inuit Tapiriit Kanatami). (2018). *National Inuit Strategy on Research*. Inuit Tapiriit Kanatami.

[CR53] Jolly, D., Berkes, F., Castleden, J., Nichols, T., & the community of Sachs Harbour. (2002). We can’t predict the weather like we used to: Inuvialuit observations of climate change, Sachs Harbour, Western Canadian Arctic. In I. Krupnik, & D. Jolly (Eds.), *The Earth is faster now: Indigenous observations of Arctic environmental change* (pp. 92–125). Arctic Research Consortium of the United States.

[CR54] Jung, T. A., Frandsen, J., Gordon, D. C. Sr., & Mossop, D. H. (2016). Colonization of the Beaufort Coastal Plain by Beaver (Castor canadensis): A Response to Shrubification of the Tundra? *The Canadian Field-Naturalist*, *130*(4). 10.22621/cfn.v130i4.1927

[CR55] Kral, M. J., & Idlout, L. (2024). Meanings of Well-Being Among Inuit in Nunavut, Canada. In F. Maggino (Ed.), Encyclopedia of Quality of Life and Well-Being Research (pp. 4213–4216). Springer. 10.1007/978-3-031-17299-1_4117

[CR56] Krause, F. (2023). Valued Volatility: Inhabiting Uncertain Flux in the Mackenzie Delta, Canada. *Social Anthropology/Anthropologie Sociale*, *31*(4), 144–173. 10.3167/saas.2023.04132306

[CR57] Kull, C. A., Kueffer, C., Richardson, D. M., Vaz, A. S., Vicente, J. R., & Honrado, J. P. (2018). Using the regime shift concept in addressing social–ecological change. *Geographical Research*, *56*(1), 26–41. 10.1111/1745-5871.12267

[CR58] Kumar, A., Devi, J. P., & Mohanasundari, T. (2025). Climate change impact assessment on livelihood vulnerability of tribal communities: a systematic review. *Mitig Adapt Strateg Glob Change*, *30*, 37. 10.1007/s11027-025-10226-9

[CR59] Lantz, T. C., Kokelj, S. V., Gergel, S. E., & Henry, G. H. (2009). Relative impacts of disturbance and temperature: Persistent changes in microenvironment and vegetation in retrogressive thaw slumps. *Global Change Biology*, *15*(7), 1664–1675. 10.1111/j.1365-2486.2009.01917.x

[CR60] Liljedahl, A. K., Boike, J., Daanen, R. P., Fedorov, A. N., Frost, G. V., Grosse, G., Hinzman, L. D., Iijma, Y., Jorgenson, J. C., Matveyeva, N., Necsoiu, M., Raynolds, M. K., Romanovsky, V. E., Schulla, J., Tape, K. D., Walker, D. A., Wilson, C. J., Yabuki, H., & Zona, D. (2016). Pan-Arctic ice-wedge degradation in warming permafrost and its influence on tundra hydrology. *Nature Geoscience*, *9*(4), 312–318. 10.1038/ngeo2674

[CR61] Lyons, N. (2010). The Wisdom of Elders: Inuvialuit Social Memories of Continuity and Change in the Twentieth Century. *Arctic Anthropology*, *47*(1), 22–38. 10.1353/arc.0.0034 20648982

[CR62] MacKerron, G. (2012). Happiness economics from 35 000 feet. *Journal of Economic Surveys*, *26*(4), 705–735. 10.1111/j.1467-6419.2010.00672.x

[CR63] Malbeuf, J. (2018). Controversial beaver management program ends early in Aklavik. CBC News, 4 June. https://www.cbc.ca/news/canada/north/beaver-management-ends-early-aklavik-1.4691106. Accessed 5 Aug 2024

[CR64] McEwen, E. H. (1953). *Report on live-trapping and transplanting of beaver in the Mackenzie Delta beaver sanctuary*. [pdf] Canadian Wildlife Service.

[CR65] Middleton, J., Cunsolo, A., Jones-Bitton, A., Shiwak, I., Wood, M., Pollock, N., Flowers, C., & Harper, S. L. (2020). We’re people of the snow: Weather, climate change, and Inuit mental wellness. *Social Science & Medicine*, *262*, 113137. 10.1016/j.socscimed.2020.113137 32889361

[CR66] Myers-Smith, I. H., Forbes, B. C., Wilmking, M., Hallinger, M., Lantz, T., Blok, D., Tape, K. D., Macias-Fauria, M., Sass-Klaassen, U., Lévesque, E., Boudreau, S., Ropars, P., Hermanutz, L., Trant, A., Siegwart Collier, L., Weijers, S., Rozema, J., Rayback, S. A., Schmidt, N. M., Schaepman-Strub, G., Wipf, S., Rixen, C., Ménard, C. B., Venn, S., Goetz, S., Andreu-Hayles, L., Elmendorf, S., Ravolainen, V., Welker, J., Grogan, P., Epstein, H. E., & Hik, D. S. (2011). Shrub expansion in tundra ecosystems: Dynamics, impacts and research priorities. *Environmental Research Letters*, *6*(4), 045509. 10.1088/1748-9326/6/4/045509

[CR67] Nagy, M. (2004). 'We did not want the muskox to increase’: Inuvialuit Knowledge about Muskox and Caribou Populations on Banks Island, Canada. In D. G. Anderson, & M. Nuttall (Eds.), *Cultivating Arctic Landscapes: Knowing and Managing Animals in the Circumpolar North* (pp. 93–109). Berghahn.

[CR68] Nagy, M. (2011). Introduction: Intellectual property and ethics. *Études/Inuit/Studies*, *35*(1–2), 7–33. 10.7202/1012833ar

[CR69] Owlijoot, P., & in consultation with the Language and Culture Committee of Nunavut Arctic College. (2008). *Guidelines for working with Inuit elders*. Nunavut Arctic College.

[CR70] Pearce, C., & Wheeler, H. C. (2026). 'Animals we’ve never seen before’: Historical wildlife management and Indigenous relations with beavers in the mainland Inuvialuit Settlement Region, Canada. *History and Anthropology*, *37*(2), 248–267. 10.1080/02757206.2025.2562809

[CR71] Pearce, T., Smit, B., Duerden, F., Kataoyak, F., Goose, A., Inuktalik, R., Ford, J., & Wandel, J. (2008). Travel routes, harvesting and climate change in Ulukhaktok, Canada. In: *Proceedings of the 4th Northern Research Forum* (pp. 56–64). Northern Research Forum.

[CR72] Pearce, T., Smit, B., Duerden, F., Ford, J. D., Goose, A., & Kataoyak, F. (2009). Inuit vulnerability and adaptive capacity to climate change in Ulukhaktok, Northwest Territories, Canada. *Polar Record*, *46*(2), 157–177. 10.1017/S0032247409008602

[CR73] Prno, J., Bradshaw, B., Wandel, J., Pearce, T., Smit, B., & Tozer, L. (2011). Community vulnerability to climate change in the context of other exposure-sensitivities in Kugluktuk, Nunavut. *Polar Research*, *30*, 7363. 10.3402/polar.v30i0.7363

[CR74] Rantanen, M., Karpechko, A. Y., Lipponen, A., Nordling, K., Hyvärinen, O., Ruosteenoja, K., Vihma, T., & Laaksonen, A. (2022). The Arctic has warmed nearly four times faster than the globe since 1979. *Communications Earth & Environment*, *3*(1), 168. 10.1038/s43247-022-00498-3

[CR75] Reo, N. J., & Ogden, L. A. (2018). Anishnaabe Aki: an indigenous perspective on the global threat of invasive species. *Sustainability Science*, *13*(5), 1443–1452. 10.1007/s11625-018-0571-4

[CR76] Reyes-García, V., García-del-Amo, D., Álvarez-Fernández, S., Benyei, P., Calvet-Mir, L., Junqueira, A. B., Labeyrie, V., Li, X., Miñarro, S., Porcher, V., & Porcuna-Ferrer, A. (2024). Indigenous Peoples and local communities report ongoing and widespread climate change impacts on local social-ecological systems. *Communications Earth & Environment*, *5*(1), 29. 10.1038/s43247-023-01164-y

[CR77] Robertson, S., & Ljubicic, G. (2019). Nunamii’luni quvianaqtuq (It is a happy moment to be on the land): Feelings, freedom and the spatial political ontology of well-being in Gjoa Haven and Tikiranajuk, Nunavut. *EPD: Society and Space*, *37*(3), 542–560. 10.1177/0263775818821129

[CR78] Rocha, J. C., Peterson, G. D., & Biggs, R. (2015). Regime shifts in the anthropocene: Drivers, risks, and resilience. *Plos One*, *10*(8), e0134639. 10.1371/journal.pone.0134639 26267896 PMC4533971

[CR79] Romanovsky, V. E., Smith, S. L., & Christiansen, H. H. (2010). Permafrost thermal state in the polar Northern Hemisphere during the international polar year 2007–2009: A synthesis. *Permafrost and Periglacial Processes*, *21*(2), 106–116. 10.1002/ppp.689

[CR80] Scheffer, M., Carpenter, S., Dakos, V., & Nes, E. V. (2015). Generic indicators of ecological resilience: Inferring the chance of a critical transition. *Annual Review of Ecology Evolution and Systematics,**46*, 145–116. 10.1146/annurev-ecolsys-112414-054242

[CR81] Scott, M. (2018). Management program aims to tackle Mackenzie Delta’s pesky beaver problem. CBC News, 28 May. https://www.cbc.ca/news/canada/north/pesky-beavers-management-program-mackenzie-delta-1.4681018. Accessed 5 Aug 2024

[CR82] Shackleton, R. T., Biggs, R., Richardson, D. M., & Larson, B. M. H. (2018). Social-ecological drivers and impacts of invasion-related regime shifts: consequences for ecosystem services and human wellbeing. *Environmental Science and Policy*, *89*, 300–314. 10.1016/j.envsci.2018.08.005

[CR83] Smejkalová, T., Edwards, M. E., & Dash, J. (2016). Arctic lakes show strong decadal trend in earlier spring ice-out. *Scientific Reports*, *6*, 38449. 10.1038/srep38449 27924914 PMC5141450

[CR84] Snook, J., Cunsolo, A., Ford, J., Furgal, C., Jones-Bitton, A., & Harper, S. L. (2022). The connection between wildlife co-management and indigenous well-being: What does the academic literature reveal? *Wellbeing Space and Society*, *3*. 10.1016/j.wss.2022.100116

[CR85] Spear, M. J., Walsh, J. R., Ricciardi, A., & Zanden, M. J. V. (2021). The invasion ecology of sleeper populations: prevalence, persistence, and abrupt shifts. *BioScience*, *71*(4), 357–369. 10.1093/biosci/biaa168

[CR86] St. Jacques, J. M., & Sauchyn, D. J. (2009). Increasing winter baseflow and mean annual streamflow from possible permafrost thawing in the Northwest Territories, Canada. *Geophysical Research Letters*, *36*(1), 6. 10.1029/2008GL035822

[CR87] Statistics Canada. (2022a). *Tables 98-10-0002-02: Population and dwelling counts: Canada, provinces and territories, and census subdivisions (municipalities)*. 10.25318/9810000201-eng

[CR88] Statistics Canada. (2022b). *Tables 98-10-0266-01: Indigenous identity by Registered or Treaty Indian status: Canada, provinces and territories, census divisions and census subdivisions.* 10.25318/9810026601-eng

[CR89] Stuckenberger, A. N. (2006). Sociality, temporality and locality in a contemporary Inuit community. *Études/Inuit/Studies*, *30*(2), 95–111. 10.7202/017567ar

[CR90] Sturm, M., Racine, C., & Tape, K. (2001). Climate change: Increasing shrub abundance in the Arctic. *Nature*, *411*, 546–547. 10.1038/35079180 11385559

[CR91] Surdu, C. M., Duguay, C. R., Brown, L. C., & Fernández Prieto, D. (2014). Response of ice cover on shallow lakes of the North Slope of Alaska to contemporary climate conditions (1950–2011): Radar remote-sensing and numerical modeling data analysis. *The Cryosphere*, *8*(1), 167–180. 10.5194/tc-8-167-2014

[CR92] Tape, K. D., Clark, J. A., Jones, B. M., Kantner, S., Gaglioti, B. V., Grosse, G., & Nitze, I. (2022). Expanding beaver pond distribution in Arctic Alaska, 1949 to 2019. *Scientific Reports*, *12*, 7123. 10.1038/s41598-022-09330-6 35504957 PMC9065087

[CR93] Tape, K. D., Gustine, D. D., Ruess, R. W., Adams, L. G., & Clark, J. A. (2016). Range expansion of moose in Arctic Alaska linked to warming and increased shrub habitat. *PLOS One*, *11*(7), e0160049. 10.1371/journal.pone.0152636 27074023 PMC4830447

[CR94] Tape, K. D., Jones, B. M., Arp, C. D., Nitze, I., & Grosse, G. (2018). Tundra be dammed: Beaver colonization of the Arctic. *Global Change Biology*, *24*(10). 10.1111/gcb.14332 29845698

[CR95] Theodorakea, I. T., & von Essen, E. (2016). Who let the wolves out? Narratives, rumors and social representations of the wolf in Greece. *Environmental Sociology*, *2*(1), 29–40. 10.1080/23251042.2015.1119349

[CR96] Turner, C. K., Lantz, T. C., & Gwich’in Tribal Council Department of Cultural Heritage. (2018). Springtime in the Delta: The socio-cultural importance of Muskrats to Gwich’in and Inuvialuit trappers through periods of ecological and socioeconomic change. *Human Ecology*, *46*, 601–611. 10.1007/s10745-018-0014-y

[CR97] Verdonen, M., Barrio, I. C., Barbero-Palacios, L., López-Blanco, E., Speed, J. D., Defourneaux, M., García Criado, M., Moullec, M. L., Mellard, J. P., Salazar, A., & Westergaard, B., K (2026). Borealization of tundra ecosystems with climate and land-use change. *Environmental Research Letters*, *21*(1), 013002. 10.1088/1748-9326/ae2e18

[CR98] Wehi, P. M., Kamelamela, K. L., Whyte, K., Watene, K., & Reo, N. (2023). Contribution of Indigenous Peoples’ understandings and relational frameworks to invasive alien species management. *People and Nature*, *5*(5), 1403–1414. 10.1002/pan3.10508

[CR99] Wilson, N. J., Walter, T. M., & Waterhouse, J. (2014). Indigenous knowledge of hydrologic change in the Yukon River Basin: A case study of ruby. *Alaska Arctic*, *68*(1), 93–106. 10.14430/arctic4459

[CR100] Wishart, R. P. (2004). A story about a Muskox: Some implications of Tetlit Gwich’in human-animal relationships. In D. G. Anderson, & M. Nuttall (Eds.), *Cultivating Arctic Landscapes: Knowing and Managing Animals in the Circumpolar North* (pp. 79–92). Berghahn.

[CR101] Xu, L., Myneni, R. B., Chapin, F. S., III, Callaghan, T. V., Pinzon, J. E., Tucker, C. J., Zhu, Z., Bi, J., Ciais, P., Tømmervik, H., Euskirchen, E. S., Forbes, B. C., Piao, S. L., Anderson, B. T., Ganguly, S., Nemani, R. R., Goetz, S. J., Beck, P. S. A., Bunn, A. G., Cao, C., & Stroeve, J. C. (2013). Temperature and vegetation seasonality diminishment over northern lands. *Nature Climate Change*, *3*(6), 581–586. 10.1038/nclimate1836

[CR102] Ziegler, J. A., Lantz, T. C., Overeem, T., Proverbs, T. A., & Lord, S. Aklavik Hunters and Trappers Committee, Gwich’in Tribal Council Department of Culture and Heritage and Inuvik Hunters and Trappers Committee. (2024). All the rivers we used to travel by: Indigenous knowledge of hydrological change and its impacts in the Mackenzie Delta Region, Canada. *Regional Environmental Change,**24*(66). 10.1007/s10113-024-02209-4

